# Students’ perceptions of need-supportive teaching, basic psychological need satisfaction, and life skills development in Chinese physical education

**DOI:** 10.3389/fpsyg.2025.1430769

**Published:** 2025-04-17

**Authors:** Xiangbo Ji, Shaofeng Zheng, Liping Cheng

**Affiliations:** ^1^School of Physical Education and Sport Science, Nanjing Normal University, Nanjing, China; ^2^Department of Physical Education, The Open University of Fujian, Fuzhou, Fujian, China; ^3^School of Physical Education and Sport Science, Fujian Normal University, Fuzhou, Fujian, China

**Keywords:** teacher need-support, basic psychological need satisfaction, life skills development, self-determination theory, PE teaching

## Abstract

**Introduction:**

Physical education (PE) is viewed as an environment conducive to students’ life skills development. However, less is known about the processes by which young people develop their life skills in PE. This is particularly the case in non-English speaking countries such as China. Based on self-determination theory, the present study aimed to investigate the relationships between students’ perceptions of teacher need-support, basic psychological need satisfaction and their life skills development in PE.

**Methods:**

The present study employed a cross-sectional design. Participants were 727 Chinese students (*M_age_* = 16.93) attending 29 classes from 7 different schools in China. They completed measures assessing these variables. The preliminary analyses used descriptive statistics to determine participants’ scores on each variable and correlations to assess the relationship between variables. Mediation analyses were conducted to evaluate the tested models.

**Results:**

In all analyses, structure, autonomy support, involvement, and total teacher need-support was positively correlated with total psychological need-satisfaction, which, in turn, positively correlated with students’ life skills development in PE. The mediation analyses showed that total psychological need-satisfaction was a key mediator between teacher need-support and students’ life skills development in PE.

**Conclusion:**

This study thus provides actionable insights into the role that teacher need-support plays in satisfying students’ basic psychological needs and, in turn, developing students’ life skills in PE. The findings highlight that teachers should exhibit need-support (e.g., provide students with constructive, clear, and self-oriented feedback; identify students’ interests, preferences and acknowledge their opinions; and develop an effective bond with students) in order to satisfy students’ three basic psychological needs and promote their life skills development in PE.

## 1 Introduction

The personal development of students is viewed as a key objective of modern Physical Education (PE) (Cronin et al., 2018). For example, in Ministry of Education of the People’s Republic of China (2022), the objective of the physical education curriculum is to enhance students’ physical and mental health. Students’ personal development can also be conceptualized via the life skills they learn (Goudas, 2010). Life skills are described as skills that people develop in one context (such as at home, school, sport) and that are also used effectively in other contexts beyond which they were learnt (Williams et al., 2022). Examples of such skills include problem solving, goal setting, social skills, and emotional skills. The importance of life skills is highlighted by the fact that they are positively correlated with young people’s health (World Health Organization, 2009), academic achievement, occupational success (Steptoe and Wardle, 2017), economic prosperity (Bailey et al., 2013), and global citizenship (UNESCO, 2016).

Along with sports, music, and drama (Larson, 2000; Holt et al., 2017), PE has been identified as a space that facilitates life skills development (Goudas, 2010). For example, past research on various forms of PE (e.g., the Sport Education Model and Cooperative Learning) has found that PE can teach students problem solving and decision making, teamwork, social skills, communication, and leadership (Dyson et al., 2004; Smither and Zhu, 2011). Moreover, when life skills programs are incorporated into PE, they can teach students how to set goals, solve problems, and make decisions (Goudas and Giannoudis, 2008; Pesce et al., 2016). Opstoel et al. (2020) recently highlighted the impact of PE on students’ development of the following life skills: work ethic, goal setting, decision making, problem solving, responsibility, leadership, cooperation, social skills, communication, and prosocial behavior.

The scholarly literature discusses four primary reasons why students develop life skills through PE. First, the interactive (i.e., students must work together), social, (i.e., it involves socializing with different students) and emotional (i.e., students must deal emotionally with success and failure) elements of PE may bring about life skills learning in students (Cronin et al., 2020). Second, the multisport nature of PE may provide students with the opportunity to develop different life skills (Goudas and Giannoudis, 2008). Third, the leadership of trained PE teachers (Pate et al., 2006) may help to create the kind of learning environment where students can acquire life skills (Opstoel et al., 2020). Fourth and finally, the teaching methods that PE instructors use in PE (e.g., cooperative learning) ought to promote the development of certain life skills.

Several propositions and models of life skills development and positive youth development (PYD) have suggested how young may learn life skills through sport and PE. At this point, it is important to highlight that sport and PE share lots of similarities (e.g., their interactive and emotional nature, their general popularity among young people) (Smith et al., 2016) and yet have some subtle differences (e.g., the sometimes compulsory nature of PE versus the voluntary nature of sports participation, and the extensive training of PE teachers versus the lesser training of volunteer sports coaches). Nonetheless, a great deal can be learnt from the below propositions and models which deal predominantly with sport, but are also of relevance to PE. These include Turnnidge et al. (2014) propositions regarding implicit and explicit life skills transfer, Bean et al. (2018) implicit/explicit continuum of life skills development and transfer, Gould and Carson (2008) model of coaching life skills through sport, Pierce et al. (2017) model for life skills transfer, and Holt et al. (2017) model of positive youth development through sport. To begin with, Turnnidge et al. (2014) discussed how both implicit and explicit processes can contribute to the development of life skills. The implicit approach involves young people learning life skills as a by-product of taking part in a sport which focuses predominantly on sport-specific skills. Conversely, the explicit approach focuses on teaching both the sport and life skills. Building upon this proposition, Bean et al. (2018) developed an implicit/explicit continuum of life skills development and transfer which included six levels: (1) structuring the sport context, (2) facilitating a positive climate (3) discussing life skills, (4) practicing life skills, (5) discussing transfer, and (6) practicing transfer. Levels 1–2 of this continuum involved the aforementioned implicit approach, whereas levels 3–6 involved the explicit approach. Taking a broader perspective, Gould and Carson (2008) developed a model explaining the wide variety of factors which impact the coaching of life skills through sport. These researchers suggested that the following factors help participants develop their life skills through sports: internal assets (e.g., existing life skills and personality characteristics); external assets (e.g., the influence of coaches, parents, and peers); sport participation experiences (e.g., the coaching philosophy and teaching strategies); and the social environment (e.g., a sense of belonging and positive social norms). More recently, Pierce et al. (2017) considered numerous factors when proposing a model for the development and transfer of life skills. This model highlighted that the following factors influence the development and transfer of participants’ life skills: the individual learner (i.e., their autobiographical experiences, internal assets, and external assets); the sport learning context (i.e., the demands of the sport, program design, coaching characteristics and strategies); and the wider socio-cultural environment. Finally, Holt et al. (2017) presented a model of positive youth development through sport by synthesizing qualitative studies. Focusing in on key factors for life skills development, these researchers suggested that life skills development can be achieved if there is a life skills program focus (i.e., life skill building and transfer activities) and a positive coach, parent and peer climate. The aforementioned propositions and models help us to understand the array of factors facilitating the learning of life skills in sports and PE. Nonetheless, a limitation of these propositions and model is their lack of a theoretical grounding. To address this limitation, self-determination theory (SDT) can help to clarify the processes by which young people learn life skills in sport and PE (Hodge et al., 2013, 2016; Ryan and Deci, 2017).

As a theory of human development, SDT (Ryan and Deci, 2017) is a useful framework for understanding how students’ learn life skills through PE. One key component of SDT is the degree to which students’ three basic psychological needs for competence, autonomy, and involvement are satisfied (Haerens et al., 2015). Competence pertains to the need to feel effective or have a sense of mastery over an activity; autonomy refers to the students’ need to self-regulate their experiences and actions; and involvement refers to the extent to which students feel socially connected and cared for by others (Ryan and Deci, 2017). These three basic psychological needs are seen as “innate psychological nutriments that are essential for ongoing psychological growth” (Deci and Ryan, 2000). So, by nurturing these three basic psychological needs in young people, it ought to allow them to develop optimally and promote their overall well-being (Ryan and Deci, 2017). Another key component of SDT is a need-supportive climate (Ryan and Deci, 2017). Within education, need-supportive teaching is defined as a teacher’s provision of structure, autonomy support, and involvement (Stroet et al., 2013). Structure includes providing a well-structured learning environment, which involves different levels of difficulty for different students, enough time for task completion; clear, constructive, and self-oriented feedback; and establishing clear expectations (Aelterman et al., 2019; Reeve et al., 2022). Autonomy support involves the teacher adopting the student’s perspective; allowing for student choice; acknowledging students’ feelings; encouraging students to solve problems and display initiative; encouraging both independent and group work; and explaining the rationale for tasks (DeMeyer et al., 2016). Involvement includes providing students with care and emotional support; demonstrating interest in students; listening empathetically; acknowledging and respecting different perspectives and feelings; encouraging cooperation and developing an affective bond with students (Reeve et al., 2022). Within SDT (Ryan and Deci, 2017; Reeve et al., 2022), structure, autonomy support and involvement are proposed to positively impact upon the three basic psychological needs, and in turn bring about positive outcomes in young people. Building on this idea, Hodge et al. (2013, 2016) have developed a conceptual model of life skills development that incorporated the key tenets of SDT. In particular, Hodge et al. (2013, 2016) suggested that a need-supportive environment should help to satisfy individuals’ basic psychological needs for autonomy, competence, and involvement, and in turn, help them to acquire life skills.

Later studies have used the SDT-based model developed by Hodge et al. (2013, 2016) to investigate life skills learning in PE. For example, Cronin et al. (2018, 2019, 2020) used SDT as their theoretical framework for studying how young people develop life skills in PE. Their first study (Cronin et al., 2018) showed that teacher autonomy support is positively related to participants’ life skills development in PE. Their second study (Cronin et al., 2019) showed that autonomy, competence, and involvement satisfaction are important mediators that explain the associations between teacher autonomy support and student’s life skills development in PE. Lastly, their third study, which was longitudinal in nature, showed that total psychological need satisfaction measured at the beginning of the school term predicted students’ life skills development at the end of the school term. It is noteworthy that the latter two studies (Cronin et al., 2019, 2020) showed that total psychological need satisfaction has the greatest impact on student’s life skills development as compared to each of the three individual needs. Overall, the above studies indicate that SDT is a reliable framework for investigating students’ learning of life skills through PE.

Building on these studies, researchers should determine whether other elements of need support (i.e., structure and involvement) are positively associated with students’ basic psychological need satisfaction and learning of life skills in PE. In this regard, Van de called for the combined use of all three dimensions of need-supportive behaviors. PE instructors rely on a wide range of teaching strategies in their daily instructional practices (Burgueño et al., 2022), so investigating only one kind of teaching strategy, such as autonomy support, limits our understanding of the relationship between other teaching strategies and life skills development. PE research has shown that structure, autonomy support, and involvement are all positively related to satisfaction of PE students’ needs for autonomy, competence, and involvement (Taylor and Ntoumanis, 2007; Standage et al., 2012). However, the relationships between structure, involvement, and total need support with life skills development has not yet been investigated. Nevertheless, consistent with the tenets of SDT (Ryan and Deci, 2017), researchers have found that students’ perceptions of teacher need-support are positively correlated with a range of variables, such as student engagement (Stroet et al., 2013; Leo et al., 2020), motivation (Stroet et al., 2013; Burgueño et al., 2022), intention to be physically active (Behzadnia, 2020; Aibar et al., 2021), and well-being (Behzadnia, 2020). Based on the above studies, it is reasonable to hypothesize that within PE, total need support, along with each of the three need-supportive behaviors (structure, autonomy support, and involvement), can be treated as antecedents of students’ life skills development. Additionally, SDT-based models (e.g., Hodge et al., 2013, 2016; Ryan and Deci, 2017) highlight that basic psychological need satisfaction ought to mediate the positive associations between teacher need-support and life skills development in PE. Specifically, teacher need support (i.e., the independent variable) should be positively associated with basic psychological need satisfaction (i.e., the mediator); which, in turn, should be positively associated with life skills development in PE (i.e., the dependent variable).

It must be noted that most research on life skills development has been conducted in English-speaking western countries. As such, it is important to research how students in other countries and cultures develop their life skills in PE (Ji et al., 2022). This line of inquiry is particularly promising as cultural differences may affect student’s learning of life skills in PE. For example, if we compare the mean scores of Chinese students’ life skills development in PE (Ji et al., 2022) with their British counterparts (Cronin et al., 2018), we see that Chinese students scored higher on total life skills development (3.66 versus 3.18 on a 1–5 point scale). Similarly, differences between countries and cultures have been seen in past SDT-based studies. For instance, Wang et al. (2021) found that teacher need-support was associated with negative affect in East-Central Europe, but not in Southeast Asia, Latin America, and Western Europe (Wang et al., 2021). Given the lack of studies on life skills development in PE across countries and cultures, it is unclear how SDT-based variables will interact with students’ life skills development in PE. The current study will therefore add to our understanding of how Chinese students develop their life skills in PE. From a practical standpoint, this study ought to help inform Chinese PE teachers how to best promote their student’s life skills development.

### 1.1 The present study

Using SDT as our theoretical framework, the purpose of this study was to investigate the relationships between student’s perceptions of teacher need-support, basic psychological need satisfaction, and life skills development in PE. Based on the findings of previous studies and the propositions of various SDT researchers (e.g., Hodge et al., 2016; Ryan and Deci, 2017; Cronin et al., 2020; Zheng et al., 2023; Howard et al., 2024; Saiz-González et al., 2024), we proposed the following three hypotheses.

Our first hypothesis was that teacher structure, autonomy support, involvement, and total need-support would be positively related to students’ basic psychological need satisfaction. Our second hypothesis was that teacher structure, autonomy support, involvement, and total need-support would be positively related to students’ development of all eight life skills in PE. Our third hypothesis was that total basic psychological need satisfaction would mediate the positive correlations between students’ perceptions of teacher structure, autonomy support, involvement, and total need-support, and their development of life skills in PE.

## 2 Methods

### 2.1 Participants

A total of 760 students from different regions of China participated in this study. However, 33 students were omitted from the final sample because they did not adequately or clearly respond to some survey items (i.e., they responded several times to the same item and/or failed to respond to numerous other items). The final sample thus included 727 students (344 males and 383 females) between 11–19 years of age (*M*_age_ = 16.93; *SD* = 2.00). The students participated in PE for an average of 1.59 h per week (*SD* = 0.24, range = 1.50–2.25) and were taking PE as an exam subject. In total, 7 schools and 29 classes were included and there was an average of 25.07 students in each class (*SD* = 5.47, range = 13–39). In PE classes, the students took part in a variety of sports including basketball, football, volleyball, badminton, table tennis, rope skipping, track and field, martial arts, and tai chi. Within the sample, 88.72% of students participated in extracurricular sports at school for an average of 1.64 h per week (*SD* = 1.84 h, range = 0.50–15.00 h). Furthermore, 23.52% of students participated in sports outside of school, for an average of 1.41 h per week (*SD* = 1.11, range = 0.50–8.33 h).

### 2.2 Measures

#### 2.2.1 Need-supportive teaching style

The 15-item Chinese version of the Need-Supportive Teaching Style Scale in Physical Education (Liu and Chung, 2017) was utilized to measure the perception that students had of teacher need-support. This 15-item scale (five items per subscale) measures three aspects of needs support: structure, autonomy support, and involvement. Example items included: “my teacher always makes it clear what he or she expects of me in class” (structure); “my teacher gives me a lot of choices” (autonomy support); and “my teacher knows me well” (involvement). Responses were provided on a 7-point scale ranging from 1 (strongly disagree) to 7 (strongly agree). The subscales of this measure demonstrated strong internal consistency reliability in previous studies with Chinese students, the Cronbach’s alpha coefficients ranged from 0.77 to 0.91 (Liu and Chung, 2017). In this study, the Cronbach’s alpha coefficients for the subscales ranged from 0.79–0.94 (see Table 1 for alpha coefficients for each subscale).

**Table 1:** Mean scores, standard deviations, reliability coefficients and interactions for all study variables.

	1	2	3	4	5	6	7	8	9	10	11	12	13
(1) Total need-support	–												
(2) Structure	0.88^**^	–											
(3) Autonomy support	0.94^**^	0.74^**^	–										
(4) Involvement	0.90^**^	0.64^**^	0.80^**^	–									
(5) Total need satisfaction	0.77^**^	0.62^**^	0.73^**^	0.73^**^	–								
(6) Teamwork	0.52^**^	0.42^**^	0.49^**^	0.48^**^	0.59^**^	–							
(7) Goal setting	0.50^**^	0.42^**^	0.50^**^	0.46^**^	0.58^**^	0.67^**^	–						
(8) Social skills	0.42^**^	0.32^**^	0.39^**^	0.45^**^	0.52^**^	0.61^**^	0.56^**^	–					
(9) Problem solving	0.45^**^	0.35^**^	0.43^**^	0.44^**^	0.51^**^	0.64^**^	0.68^**^	0.60^**^	–				
(10) Emotional skill	0.41^**^	0.36^**^	0.39^**^	0.36^**^	0.50^**^	0.55^**^	0.56^**^	0.49^**^	0.60^**^	–			
(11) Leadership	0.45^**^	0.34^**^	0.42^**^	0.45^**^	0.54^**^	0.68^**^	0.64^**^	0.69^**^	0.68^**^	0.60^**^	–		
(12) Time management	0.42^**^	0.34^**^	0.40^**^	0.40^**^	0.47^**^	0.59^**^	0.64^**^	0.52^**^	0.62^**^	0.58^**^	0.72^**^	–	
(13) Communication	0.44^**^	0.38^**^	0.40^**^	0.39^**^	0.48^**^	0.57^**^	0.50^**^	0.59^**^	0.57^**^	0.56^**^	0.65^**^	0.55^**^	–
Scale range	1–7	1–7	1–7	1–7	1–7	1–5	1–5	1–5	1–5	1–5	1–5	1–5	1–5
Mean score	5.16	5.47	5.16	4.85	4.98	3.51	3.44	3.44	3.51	3.55	3.33	3.31	3.68
Standard deviation	1.00	1.10	1.12	1.08	0.98	0.69	0.80	0.79	0.75	0.76	0.71	0.81	0.71
Cronbach’s alpha	0.94	0.79	0.92	0.91	0.91	0.90	0.93	0.88	0.89	0.84	0.89	0.88	0.84

*N* = 727. **p < 0.01. Problem solving, problem solving and decision making; Communication, interpersonal communication skills.

#### 2.2.2 Basic psychological need satisfaction

The Chinese version of the Psychological Need Satisfaction Scale for PE (Liu and Chung, 2014) was used to assess students’ psychological need satisfaction. The item stem for the scale was: “In my physical education classes, I...” Items measuring three factors were included: autonomy (e.g., “have opportunities to express my views and thoughts”), competence (e.g., “get opportunities to feel that I am good at sports/PE”), and involvement (e.g., “feel comfortable when being with people”). Participants responded to the items using a 7-point scale (1 = *strongly disagree*, 7 = *strongly agree*). This measure has demonstrated a high degree of internal consistency reliability in previous studies with Chinese students, the Cronbach’s alpha coefficients ranged from 0.83 to 0.87 (Liu and Chung, 2014). As in past studies (e.g., Behzadnia, 2020; Leo et al., 2020), the current study combined the three needs to create a total psychological need satisfaction score. Previous research has shown that total psychological need satisfaction was more important for life skills development in PE than each of the three individual needs in isolation (Cronin et al., 2019, 2020). In this study, the Cronbach’s alpha coefficients for the total psychological need satisfaction subscale was 0.91.

#### 2.2.3 Life skills development

The 43-item Chinese version of Life Skills Scale for PE (Ji et al., 2022) was used to measure how students perceived their own life skills development. The item stem for this scale was “PE classes have taught me to....” Example items included teamwork (7 items; e.g., “work well in a team/small group”); goal setting (7 items; e.g., “set goals so I can focus on improving”); social skills (5 items; e.g., “initiate a conversation”); problem solving and decision making (4 items; e.g., “be able to think carefully about a problem”); emotional skills (4 items, e.g., “know how to deal with emotions”); leadership (8 items; e.g., “know how to positively influence a group of people”); time management (4 items; e.g., “assess how much time I spend on various activities”); and interpersonal communication (4 items; e.g., “talking clearly to others”). The students provided responses to the items using a 5-point Likert scale, ranging from 1 (“not at all”) to 5 (“very much”). In previous studies conducted with Chinese students, Cronbach’s alpha coefficients for the life skills subscales ranged from 0.80 to 0.96, indicating strong internal consistency and reliability (Ji et al., 2022). In this study, Cronbach’s alpha coefficients for the life skills subscales ranged from 0.84 to 0.93.

### 2.3 Procedures

When recruiting participants for the study, the first author contacted the PE department at the students’ schools to obtain approval for data collection. Our inclusion criteria for the study included middle school, high school, and first-year college students who regularly participate in PE classes. In China, the age range of students in these schools is usually 12–15 years old for middle school students; 16–18 years old for high school students; and 19–22 years old for college students. It is worth noting that in some cases there may be a discrepancy between the age of the student and their period of study, for instance, if they started school early, late or repeated a year. Each student provided written informed consent before participating in the study, and students who are minors also obtained written informed consent from their parents. Specifically, prior to the start of the study, the first author prepared a written informed consent detailing the study purpose, study procedure, time and place of participation, potential benefits and risks, and clearly stating that participants have the right to access their own data, request correction or deletion of the same data, and can withdraw from the study at any time without consequences. At the same time, leave enough space for participants and their legal guardians to sign and date to indicate that they have read and understood the content, and finally add the email address and phone number of the first author, telling them to contact them if they have any questions or concerns about the study. As for the signing details of the informed consent, the first author explained the contents of the informed consent face-to-face with the participants during the school period, gave them enough time to ask questions, patiently answered their questions, and ensured that they truly understood the relevant information of the study. After returning home from school, participants will bring the informed consent to the legal guardian. After the legal guardian agrees to participate in the study and signs, they will be collected. During the study, the first author maintained an open line of communication to ensure that participants and their legal guardians received timely answers and assistance if they had any questions or concerns about the study. At the end of the study, we report the results of the study to the participants and their legal guardians in the form of a written report, so that they understand the results of the study, and at the same time, re-emphasize their rights and contributions during the study, as well as their right to withdraw from the study at any time. The signing process and details are the same as the informed consent form. The questionnaires were filled out during their physical education class or self-study sessions to ensure that students were fully exposed to the variables investigated in the study. Most participants took about 15–20 min to complete the survey.

At the time of data collection, each participant was anonymously coded using randomly generated alphanumeric combinations. The raw data is stored on an institutional encrypted server with access only to the first and corresponding authors, and enhance login security with biometrics and dynamic tokens. At the same time, to prevent data loss or damage, the backup data is backed up using an external hard disk, and data restoration tests are performed periodically to ensure the availability of the backup data. During data entry, format check and logical verification are carried out on the input data to prevent illegal or incorrect data from entering the system. During data transmission, a checksum digital signature is used to verify whether the data is tampered with during transmission. Finally, 5 years after the end of the study, the data will be physically destroyed (paper) and cryptographically erased (electronic).

To be clear, our study was exempt from ethical scrutiny. Because our research is in line with the regulations on exemption from ethical review in China’s “Measures for Ethical Review of Life Science and Medical Research Involving Human Subjects” issued in 2023. That is, through a simple anonymous questionnaire survey, research that does not involve in-depth exploration of human physiological and psychological behavior, does not use new technologies and new products to conduct human experiments, does not cause harm to the human body, and does not involve sensitive personal information or commercial interests can be exempted from ethical review (National Health Commission, Ministry of Education, Ministry of Science and Technology, State Administration of Traditional Chinese Medicine et al., 2023).

### 2.4 Statistical analyses

SPSS Version 25.0 (IBM Corporation, 2017) was used for our preliminary analyses and descriptive statistics, as well as to calculate Pearson’s correlations (a parametric test) between the study variables. MLwiN Version 3.01 (Rasbach et al., 2017) was used to determine whether multilevel analyses would be appropriate for our data analyses. Specifically, we calculated intraclass correlation coefficients (ICCs) for all variables at both the school and the class levels. ICCs greater than 0.10 (Preacher et al., 2011) indicate that a considerable portion of the variance is at the school or class level and thus that multilevel modelling is appropriate. For correlation analysis, all variables in this study are continuous variables, and the sample size is much larger than 30; therefore, Pearson correlation analysis is suitable for this study (Mather, 2012). For the mediation analyses, we first determined whether there were significant correlations among our predictor, mediator, and criterion variables before proceeding with our analyses. When conducting the mediation analyses, we used model number four of the PROCESS macro for SPSS (Hayes, 2013) with 20,000 bootstrap resamples and a 95% bias corrected confidence interval (CI). This analysis takes into account both direct and indirect effects and performs better than other techniques in terms of statistical power and Type I error control (Hayes, 2009). Following Mathieu and Taylor (2006) distinction between mediation and indirect effects, we first determined whether mediation was occurring before assessing the indirect effect of each potential mediator. Mediation occurs when a statistically significant regression coefficient (*p* < 0.05) for the total effect reduces in value for the direct effect when the mediators are entered into the model. There is thus evidence of an indirect effect when zero is not included within the lower and upper bound CIs. In terms of the effect sizes within our analyses, correlations were deemed small (*r* = ± 0.10 to ±0.29), medium (*r* = ± 0.30 to ±0.49), or large (*r* > ± 0.50) based on Cohen (1988) criteria. *R*^2^ values for each mediation model were also converted to Cohen’s *f*^2^ (an effect size measure) using the following formula (*R*^2^/(1 - R^2^)) and can likewise be identified as small (*f*^2^ ≥ 0.02), medium (*f*^2^ ≥ 0.15), or large (*f*^2^ ≥ 0.35) based on Cohen (1988) guidelines.

## 3 Results

### 3.1 Preliminary analyses

To assess normality, both skewness and kurtosis values were calculated for the study variables. The data showed reasonable normality as the skewness values ranged from −0.16 to −0.67, and kurtosis values ranged from −0.02 to 1.45 (Tabachnick and Fidell, 2013). Our analyses to assess if multilevel modeling would be appropriate revealed that the mean ICC at the school-level was 0.01 (*Range* = 0–0.05, *SD* = 0.01), and at the class-level was 0.05 (*Range* = 0.02–0.10, *SD* = 0.02). As these values are lower than the threshold for multilevel analysis to be appropriate (Preacher et al., 2011), we proceeded with our analyses only at the individual level.

### 3.2 Descriptive statistics

Table 1 displays the mean scores, standard deviations, reliability coefficients, and intercorrelations for all variables. The mean scores for teacher need support (structure, autonomy support, and involvement) on the 1–7 response scales ranged from 4.85 to 5.47, and the mean score for total need satisfaction was 5.16. The mean scores for the eight life skills on the 1–5 response scales ranged from 3.31 to 3.68. The correlations showed that total need support and the three components of need support (i.e., structure, autonomy support, and involvement) were positively correlated with students’ total basic psychological need satisfaction (*r* range = 0.62 to 0.77, all *p* values <0.01). Total need support and the three components of need support were also positively correlated with students’ development of the eight life skills (*r* range = 0.32 to 0.52, all *p* values <0.01). Finally, total need satisfaction was positively associated with students’ development of the eight life skills (*r* range = 0.47 to 0.59, all *p* values <0.01). The results of the correlational analysis indicated that we can proceeded to conduct further mediation analyses.

### 3.3 Mediation analyses

**Figure 1:**
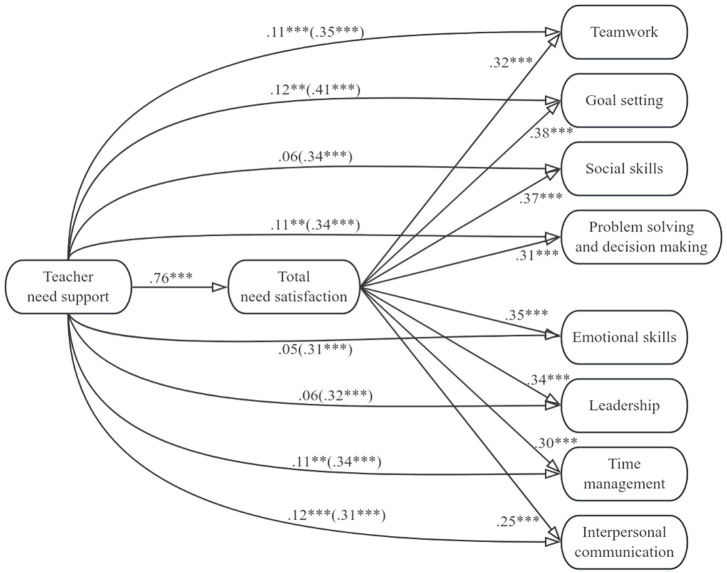
Model including total need support and the eight life skills. Values signify unstandardized regression coefficients. The direct effect of total need support on each of the life skills is outside the parentheses, whereas the total effect is inside the parentheses. **p < 0.01, ***p < 0.001.

**Figure 2:**
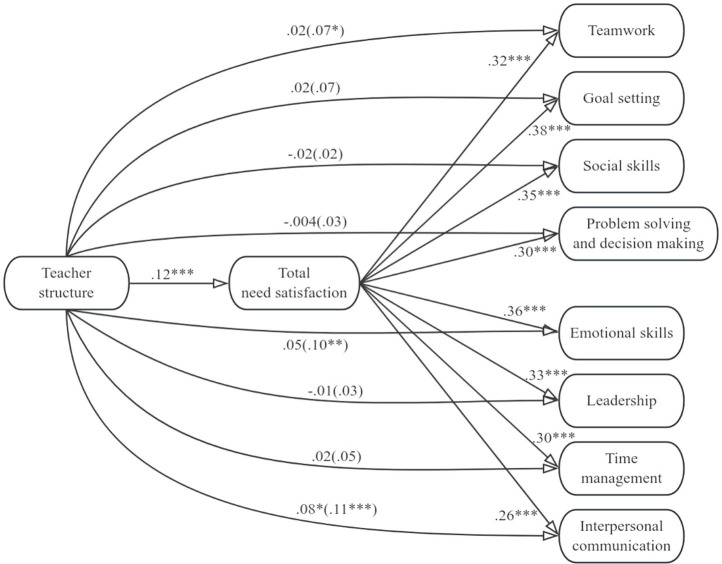
Model including teacher structure and the eight life skills. Values signify unstandardized regression coefficients. The direct effect of teacher structure on each of the life skills is outside the parentheses, whereas the total effect is inside the parentheses. Teacher autonomy support and involvement were included as covariates. *p < 0.05, **p < 0.01, ***p < 0.001.

**Figure 3:**
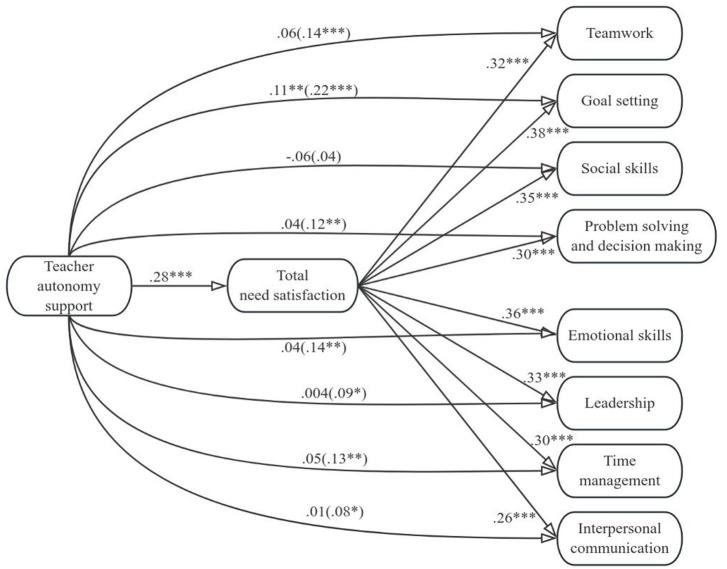
Model including teacher autonomy support and the eight life skills. Values signify unstandardized regression coefficients. The direct effect of teacher autonomy support on each of the life skills is outside the parentheses, whereas the total effect is inside the parentheses. Teacher structure and involvement were included as covariates. *p < 0.05, **p < 0.01, ***p < 0.001.

**Figure 4:**
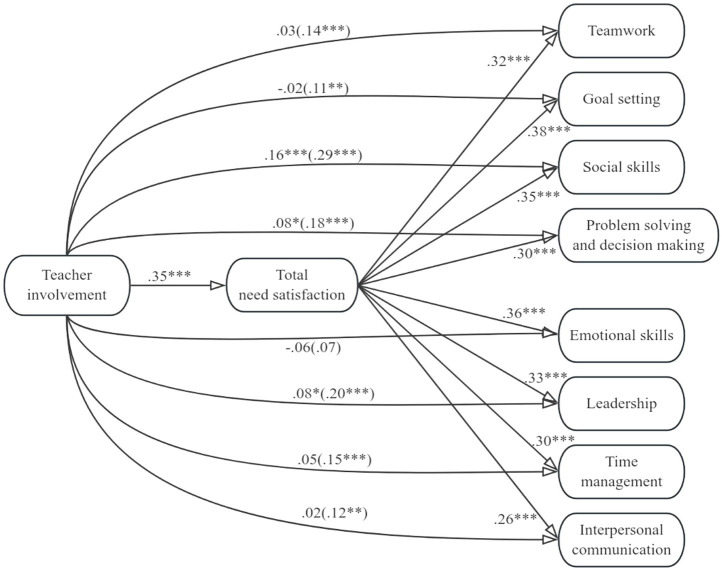
Model including teacher involvement and the eight life skills. Values signify unstandardized regression coefficients. The direct effect of teacher involvement on each of the life skills is outside the parentheses, whereas the total effect is inside the parentheses. Teacher structure and autonomy support were included as covariates. *p < 0.05, **p < 0.01, ***p < 0.001.

Figures 1–4 display the mediation models that we tested. In the four models, either total need support, structure, autonomy support, or involvement was included as the predictor variable, and total need satisfaction was included as the mediator variable. Teamwork, goal setting, social skills, problem solving and decision making, emotional skills, leadership, time management, and interpersonal communication were included as criterion variables. Tables 2–5 contain the indirect effects of total need support, structure, autonomy support, and involvement on the eight life skills through the mediator variable.

**Table 2:** Indirect effects of total need support on participants’ development of each of the life skills through total need satisfaction.

	Bootstrap effect	Bootstrap SE	95% CI
Teamwork			
Indirect effect	0.24	0.03	[0.19, 0.30]
Model	*F*(2, 724) = 198.89***, *R*^2^ = 0.35, Cohen’s *f*^2^ = 0.54		
Goal setting			
Indirect effect	0.29	0.03	[0.22, 0.35]
Model	*F*(2, 724) = 189.44***, *R*^2^ = 0.34, Cohen’s *f*^2^ = 0.52		
Social skills			
Indirect effect	0.28	0.04	[0.21, 0.35]
Model	*F*(2, 724) = 133.17***, *R*^2^ = 0.27, Cohen’s *f*^2^ = 0.37		
Problem solving			
Indirect effect	0.23	0.03	[0.17, 0.29]
Model	*F*(2, 724) = 134.39***, *R*^2^ = 0.27, Cohen’s *f*^2^ = 0.37		
Emotional skills			
Indirect effect	0.26	0.03	[0.20, 0.33]
Model	*F*(2, 724) = 121.32***, *R*^2^ = 0.25, Cohen’s *f*^2^ = 0.33		
Leadership			
Indirect effect	0.26	0.03	[0.20, 0.32]
Model	*F*(2, 724) = 147.04***, *R*^2^ = 0.29, Cohen’s *f*^2^ = 0.41		
Time management			
Indirect effect	0.23	0.04	[0.16, 0.30]
Model	*F*(2, 724) = 109.27***, *R*^2^ = 0.23, Cohen’s *f*^2^ = 0.30		
Communication			
Indirect effect	0.19	0.03	[0.13, 0.25]
Model	*F*(2, 724) = 114.79***, *R*^2^ = 0.24, Cohen’s *f*^2^ = 0.32		

*N* = 727. ***p < 0.001. 20,000 bootstrap resamples and 95% bias corrected confidence intervals were used. Cl, confidence interval. Problem solving, problem solving and decision making; Communication, interpersonal communication skills.

**Table 3:** Indirect effects of teacher structure on participants’ development of each of the life skills through total need satisfaction.

	Bootstrap effect	Bootstrap SE	95%CI
Teamwork			
Indirect effect	0.04	0.01	[0.02, 0.07]
Model	*F*(4, 722) = 99.27***, *R*^2^ = 0.35, Cohen’s *f*^2^ = 0.54		
Goal setting			
Indirect effect	0.05	0.01	[0.02, 0.08]
Model	*F*(4, 722) = 95.78***, *R*^2^ = 0.35, Cohen’s *f*^2^ = 0.54		
Social skills			
Indirect effect	0.04	0.01	[0.02, 0.08]
Model	*F*(4, 722) = 70.99***, *R*^2^ = 0.28, Cohen’s *f*^2^ = 0.39		
Problem solving			
Indirect effect	0.04	0.01	[0.02, 0.06]
Model	*F*(4, 722) = 67.92***, *R*^2^ = 0.27, Cohen’s *f*^2^ = 0.37		
Emotional skills			
Indirect effect	0.04	0.01	[0.02, 0.08]
Model	*F*(4, 722) = 62.01***, *R*^2^ = 0.26, Cohen’s *f*^2^ = 0.35		
Leadership			
Indirect effect	0.04	0.01	[0.02, 0.07]
Model	*F*(4, 722) = 74.60***, *R*^2^ = 0.29, Cohen’s *f*^2^ = 0.41		
Time management			
Indirect effect	0.04	0.01	[0.02, 0.07]
Model	*F*(4, 722) = 54.62***, *R*^2^ = 0.23, Cohen’s *f*^2^ = 0.30		
Communication			
Indirect effect	0.03	0.01	[0.02, 0.06]
Model	*F*(4, 722) = 57.82***, *R*^2^ = 0.24, Cohen’s *f*^2^ = 0.32		

*N* = 727. ***p < 0.001. Teacher autonomy support and involvement were entered as covariates in each model. 20,000 bootstrap resamples and 95% bias corrected confidence intervals were used. Cl, confidence interval; Problem solving, problem solving and decision making; Communication, interpersonal communication skills.

**Table 4:** Indirect effects of teacher autonomy support on participants’ development of each of the life skills through total need satisfaction.

	Bootstrap effect	Bootstrap SE	95%CI
Teamwork			
Indirect effect	0.09	0.02	[0.06, 0.12]
Model	*F*(4, 722) = 99.27***, *R*^2^ = 0.35, Cohen’s *f*^2^ = 0.54		
Goal setting			
Indirect effect	0.11	0.02	[0.07, 0.15]
Model	*F*(4, 722) = 95.78***, *R*^2^ = 0.35, Cohen’s *f*^2^ = 0.54		
Social skills			
Indirect effect	0.10	0.02	[0.06, 0.14]
Model	*F*(4, 722) = 70.99***, *R*^2^ = 0.28, Cohen’s *f*^2^ = 0.39		
Problem solving			
Indirect effect	0.08	0.02	[0.05, 0.12]
Model	*F*(4, 722) = 67.92***, *R*^2^ = 0.27, Cohen’s *f*^2^ = 0.37		
Emotional skills			
Indirect effect	0.10	0.02	[0.06, 0.14]
Model	*F*(4, 722) = 62.01***, *R*^2^ = 0.26, Cohen’s *f*^2^ = 0.35		
Leadership			
Indirect effect	0.09	0.02	[0.06, 0.13]
Model	*F*(4, 722) = 74.60***, *R*^2^ = 0.29, Cohen’s *f*^2^ = 0.41		
Time management			
Indirect effect	0.08	0.02	[0.05, 0.12]
Model	*F*(4, 722) = 54.62***, *R*^2^ = 0.23, Cohen’s *f*^2^ = 0.30		
Communication			
Indirect effect	0.07	0.02	[0.04, 0.10]
Model	*F*(4, 722) = 57.82***, *R*^2^ = 0.24, Cohen’s *f*^2^ = 0.32		

*N* = 727. ***p < 0.001. Teacher structure and involvement were entered as covariates in each model. 20,000 bootstrap resamples and 95% bias corrected confidence intervals were used. Cl, confidence interval; Problem solving, problem solving and decision making; Communication, interpersonal communication skills.

**Table 5:** Indirect effects of teacher involvement on participants’ development of each of the life skills through total need satisfaction.

	Bootstrap effect	Bootstrap SE	95%CI
Teamwork			
Indirect effect	0.11	0.02	[0.08, 0.15]
Model	*F*(4, 722) = 99.27***, *R*^2^ = 0.35, Cohen’s *f*^2^ = 0.54		
Goal setting			
Indirect effect	0.13	0.02	[0.09, 0.18]
Model	*F*(4, 722) = 95.78***, *R*^2^ = 0.35, Cohen’s *f*^2^ = 0.54		
Social skills			
Indirect effect	0.12	0.02	[0.08, 0.17]
Model	*F*(4, 722) = 70.99***, *R*^2^ = 0.28, Cohen’s *f*^2^ = 0.39		
Problem solving			
Indirect effect	0.10	0.02	[0.07, 0.15]
Model	*F*(4, 722) = 67.92***, *R*^2^ = 0.27, Cohen’s *f*^2^ = 0.37		
Emotional skills			
Indirect effect	0.13	0.02	[0.09, 0.17]
Model	*F*(4, 722) = 62.01***, *R*^2^ = 0.26, Cohen’s *f*^2^ = 0.35		
Leadership			
Indirect effect	0.12	0.02	[0.08, 0.16]
Model	*F*(4, 722) = 74.60***, *R*^2^ = 0.29, Cohen’s *f*^2^ = 0.41		
Time management			
Indirect effect	0.11	0.02	[0.07, 0.15]
Model	*F*(4, 722) = 54.62***, *R*^2^ = 0.23, Cohen’s *f*^2^ = 0.30		
Communication			
Indirect effect	0.09	0.02	[0.06, 0.13]
Model	*F*(4, 722) = 57.82***, *R*^2^ = 0.24, Cohen’s *f*^2^ = 0.32		

*N* = 727. ***p < 0.001. Teacher structure and autonomy support were entered as covariates in each model. 20,000 bootstrap resamples and 95% bias corrected confidence intervals were used. Cl, confidence interval; Problem solving, problem solving and decision making; Communication, interpersonal communication skills.

For the models in Figure 1, we can see that total need support was positively related to total need satisfaction, and total need satisfaction was positively related to all eight life skills. Total need support was positively correlated with all eight life skills (i.e., the total effects were significant and positive). When total need satisfaction was included as the mediator, the direct effect of total need support on social skills, emotional skills, and leadership was not statistically significant. Though reduced, however, the direct effect of total need support on teamwork, goal setting, problem solving and decision making, time management, and interpersonal communication, was still statistically significant. These results indicate that total need satisfaction mediated the correlation between total need support and participants’ development of the eight life skills. From Table 2, for all models, we can thus see that zero was not included within the lower and upper bound Cis for total needs satisfaction, suggesting need support had an indirect effect on all eight life skills via total needs satisfaction.

For the models in Figure 2, we can see that teacher structure was positively correlated with total need satisfaction and that total need satisfaction was positively associated with all eight life skills. However, teacher structure was only positively associated with teamwork, emotional skills, and international communication (i.e., the total effects were significant and positive). When total need satisfaction was included as the mediator between teacher structure and teamwork, emotional skills, and international communication, however, the direct effect of teacher structure on teamwork and emotional skills was not statistically significant, whereas the direct effect of teacher structure on interpersonal communication, though reduced, was still statistically significant. These results indicate that total need satisfaction mediated the associations between teacher structure and participants’ development of teamwork, emotional skills, and interpersonal communication. From Table 3, for all models, we can thus see that zero was not included within the lower and upper bound Cis for total needs satisfaction, suggesting teacher structure had an indirect effect on all eight life skills via total needs satisfaction.

For the models in Figure 3, we can see that teacher autonomy support was positively correlated with total need satisfaction and that total need satisfaction was positively correlated with all eight life skills. Teacher autonomy support was positively associated with students’ development of life skills besides social skills (i.e., the total effects were significant and positive). When total need satisfaction was included as the mediator between teacher autonomy support and the life skills besides social skills, the direct effect of teacher autonomy support on teamwork, problem solving and decision making, emotional skills, leadership, time management, and interpersonal communication was not statistically significant. Though reduced, however, the direct effect of teacher autonomy support on goal setting was still statistically significant. These results indicate that total need satisfaction mediated the positive correlation between teacher autonomy support and students’ development of teamwork, problem solving and decision making, emotional skills, leadership, time management, interpersonal communication, and goal setting. From Table 4, for all models, we can thus see that zero was not included within the lower and upper bound Cis for total needs satisfaction, suggesting teacher autonomy support had an indirect effect on all eight life skills via total needs satisfaction.

For the models in Figure 4, we can see that teacher involvement was positively correlated with total need satisfaction and that total need satisfaction was positively correlated with all eight life skills. Teacher involvement was positively associated with students’ development of life skills besides emotional skills (i.e., the total effects were significant and positive). When total need satisfaction was included as the mediator between teacher involvement and the life skills besides emotional skills, the direct effect of teacher involvement on teamwork, goal setting, time management, and interpersonal communication was not statistically significant, whereas the direct effect of teacher involvement on social skills, problem solving and decision making, and leadership, was still statistically significant although reduced. These results indicate that total need satisfaction mediated the relationship between teacher involvement and students’ development of teamwork, goal setting, time management, interpersonal communication, social skills, problem solving and decision making, and leadership. From Table 5, for all models, we can thus see that zero was not included within the lower and upper bound Cis for total needs satisfaction, suggesting teacher involvement had an indirect effect on all eight life skills via total needs satisfaction.

## 4 Discussion

The overall aim of the current study was to investigate how Chinese PE students develop their life skills in PE using SDT (Ryan and Deci, 2017) as our theoretical framework. Specifically, we addressed the call for the following SDT-based relationships to be investigated: need support - > basic psychological need satisfaction - > life skills development (Cronin et al., 2020). Our study also responds to the call to integrate the SDT-based concepts of a need-supportive environment and satisfaction of the three basic psychological needs into research on life skills development (Hodge et al., 2013, 2016). The novelty of this study lies in the fact that it investigated total need support, along with structure and involvement, in relation to student’s life skills development in PE, whereas past studies only focused on teacher autonomy support (e.g., Cronin et al., 2019, 2020).

Our first hypothesis was that teacher structure, autonomy support, involvement, and total need-support would be positively related to students’ basic psychological need satisfaction. The findings from our mediation analyses demonstrated that teacher structure, autonomy support, involvement and total need support were positively associated with participants’ basic psychological need satisfaction. This finding confirmed our first hypothesis and is consistent with the SDT-based proposition that structure, autonomy support, involvement, and total need support will positively impact upon the three basic psychological needs (Hodge et al., 2013, 2016; Ryan and Deci, 2017; Reeve et al., 2022). Moreover, it replicates the previous findings in PE which have shown that structure, autonomy support, involvement, and total need support were positively related to students’ basic psychological needs (Taylor and Ntoumanis, 2007; Standage et al., 2012). Overall, our findings suggest that PE teachers should engage in need-supportive teaching behaviors in order to satisfy their students three basic psychological needs. Teachers can provide structure by providing a well-structured learning environment; tasks of different difficulty levels depending on the student’s ability, enough time for task completion; clear, constructive, and individualized feedback; and establishing clear expectations (Aelterman et al., 2019; Reeve et al., 2022). Autonomy support involves the teacher listening to the student’s perspective; providing choice in activities; acknowledging students’ feelings; encouraging problem solving and initiative; utilizing independent and group work; and explaining the rationale for tasks (DeMeyer et al., 2016). Teachers can provide by being caring, interested in students, and emotionally supportive; listening empathetically; acknowledging and respecting different perspectives and feelings; encouraging teamwork and developing positive relationships with students (Reeve et al., 2022). It is important to note that not all of these behaviors are measured by the Need-Supportive Teaching Style Scale in Physical Education (NSTSSPE) (Liu and Chung, 2017) which was used in the present study. Therefore, future studies could assess how different measures of teacher need-support (which include different aspects of need support) are associated with student’s life skills development in PE.

Our second hypothesis was that teacher structure, autonomy support, involvement, and total need-support would be positively related to students’ development of all eight life skills in PE. Firstly, the findings from our mediation analyses demonstrated that teacher structure was positively associated with student’s development of teamwork, emotional skills, and interpersonal communication. Secondly, our findings showed that teacher autonomy support was positively associated with student’s development of all eight life skills. Thirdly, the findings from our mediation analyses demonstrated that involvement was positively associated with student’s development of all eight life skills. Fourthly, our findings showed that total need support was positively associated with student’s development of all eight life skills. Overall, with the exception of the lack of relationships between teacher structure and 5/8 life skills, the findings supported our hypothesis that teacher structure, autonomy support, involvement, and total need support would be positively associated with student’s development of all eight life skills in PE. Interestingly, our finding that teacher structure was only associated with 3/8 life skills suggests that teacher autonomy support and involvement are more important behaviors for teachers to display in order to bring about life skills development in their students. This finding supports Bean et al. (2018) continuum which suggests that structuring the sports environment has the least effect on young people’s life skills development as compared to other approaches. In the future, researchers should further investigate why teacher’s need supportive behaviors are positively related to their student life skills development in PE. This may involve qualitative research methods to unpick some of the positive relationships which we have found in the current study.

Our third hypothesis was that total basic psychological need satisfaction would mediate the positive correlations between students’ perceptions of teacher structure, autonomy support, involvement, and total need-support, and their development of life skills in PE. Firstly, findings from our mediation analyses indicated that total basic psychological need satisfaction mediated the positive associations between teacher structure and student’s development of teamwork, emotional skills, and interpersonal communication. To the best of our knowledge, this was the first study to illustrate that student’s basic psychological need satisfaction mediates the positive associations between teacher structure and the development of these life skills in PE. Secondly, our findings demonstrated that total basic psychological need satisfaction mediated the positive associations between teacher autonomy support and student’s development of all eight life skills (except for social skills). Such a finding replicated the studies of Cronin et al. (2019) with British PE students and supported the cross-cultural universality and applicability of SDT (Wang et al., 2021). Thirdly, findings from our mediation analyses indicated that basic psychological need satisfaction mediated the positive associations between involvement and student’s development of all eight life skills (except for emotional skills). Again, to the best of our knowledge, this was the first study to highlight that student’s basic psychological need satisfaction mediates the positive associations between involvement and the development teamwork, goal setting, social skills, problem solving and decision making, leadership, time management, and interpersonal communication. Fourthly, our findings indicated that basic psychological need satisfaction mediated the positive associations between total need support and student’s development all eight life skills. Overall, given that mediation was evident in 25/32 relationships we examined, we concluded that the findings partially supported our third hypothesis. It is difficult to speculate why 7/32 relationships we examined did not support the presence of mediation. As such, future studies may seek to replicate our findings (i.e., the lack of mediation in these instances) and further investigate such findings via qualitative research methods. Related to our mediation findings, it is important to note that there is a distinction between mediation and indirect effects, and indirect effects can take place without mediation occurring (Hayes, 2013), as was the case in our own study. In sum, our overall findings supported Hodge et al. (2013, 2016) conceptual model of life skills development which suggested that a need-supportive environment should help to satisfy individuals’ basic psychological needs and, in turn, help them to acquire life skills. Moreover, our findings were in line with previous studies in PE, which have found that basic psychological need satisfaction mediated the relationships between teacher need support and students’ engagement (Stroet et al., 2013), motivation (Haerens et al., 2015), intention to be physically active and well-being (Behzadnia, 2020). The findings from our study also provided support for level one and two of the continuum of life skills development (Bean et al., 2018), which highlights that creating an appropriately structured context (e.g., setting rules and designing a well-structured learning context–level 1) and facilitating a positive climate (e.g., fostering positive relationships, supporting efficacy and mattering–level 2) will allow participants to implicitly develop their life skills. Furthermore, our findings support the strategies for life skills development outlined in the models of Gould and Carson (2008) and Pierce et al. (2017). Such strategies include providing clear and consistent rules and instructions (i.e., structure); providing opportunities for decision making, initiative and leadership (i.e., autonomy support); developing strong coach-athlete relationships and showing interest in athletes (i.e., involvement). In practice, our findings demonstrate that teachers should aim to create a comprehensive need-supportive environment that helps to satisfy students’ three basic psychological needs and, in turn, develop their life skills.

Notably, our overall findings highlight the importance of total need support and total psychological need satisfaction for students to develop life skills in PE. This provides support for Hodge et al. (2013, 2016) model which suggests that the combined satisfaction of the three basic psychological needs is important for people to develop their life skills. Similarly, Deci and Ryan (2012) claimed that a balance between all three basic psychological needs is required for positive psychological development to occur. Our research in China, along with previous cross-sectional (Cronin et al., 2019) and longitudinal studies (Cronin et al., 2020) in Britain, support this claim within PE. Specifically, our study indicates that total need support and total psychological need satisfaction are important variables in a student’s development of the following life skills: teamwork, goal setting, social skills, problem solving and decision making, emotional skills, leadership, time management, and interpersonal communication. As such, although our study has highlighted the importance of each of the three aspects of need support, PE teachers should ideally take a holistic approach by focusing on all aspects of need support and basic psychological need satisfaction when looking to develop their students’ life skills.

## 5 Limitations and future directions

The current study provided some novel and actionable findings but had several limitations that need to be discussed. First, students’ self-reports were used, however, the accuracy and social desirability of their responses limit the effectiveness of this approach (Donaldson and Grant-Vallone, 2002; Brenner and DeLamater, 2014). Specifically, respondents may score higher on reported life skills in surveys than on actual behaviors, resulting in lower questionnaire measurement validity (Shephard, 2003; Brenner and DeLamater, 2014). Given that student, teacher and observed reports of SDT variables can differ (Aelterman et al., 2014), future studies should utilize independent classroom observers to corroborate students’ ratings of SDT-based variables, or, alternatively, they should assess life skills development through the ratings provided by parents, peers, and teachers (Cronin et al., 2019). A second limitation of our cross-sectional study is that it does not allow for causal interpretations of the findings. Specifically, it is difficult for this research method to probe into the reasons and trends of PE teachers’ need support behaviors promoting the development of students’ life skills. As such, building on our initial positive findings, future SDT-based studies on life skills development in PE should utilize both longitudinal and experimental research designs. Third, Third, we only focused on teacher need-support but not on need-thwarting teaching practices, only on basic psychological need satisfaction but not on basic psychological need frustration, and only on total basic psychological need satisfaction and not on satisfaction of each of the three basic psychological needs. As such, future studies could also investigate need thwarting teaching behaviors, basic psychological need frustration, and satisfaction of each of the three basic psychological needs when investigating life skills development in PE.

## 6 Conclusion

The present study extended previous findings in PE by showing that students’ perceptions of structure, autonomy support, involvement and total need support are associated with Chinese students’ life skills development in PE. Grounded in SDT, our findings showed that students’ perceptions of teacher structure, autonomy support, involvement and total need support are positively correlated with their development of eight different life skills in PE and that basic psychological need satisfaction mediates the majority of these correlations. These findings point the way for future studies to investigate PE students’ life skills development via SDT. The current findings also suggest that there is a need to train PE teachers in the benefits of using SDT-based principles for developing students’ life skills. From an applied perspective, the findings highlight the importance of promoting a well-structured, autonomy supportive, and involving PE environment for promoting students’ life skills development in PE.

## Data Availability

The original contributions presented in the study are included in the article/supplementary material, further inquiries can be directed to the corresponding author/s.
